# Insulin-Like Growth Factor Binding Proteins and IGFBP Proteases: A Dynamic System Regulating the Ovarian Folliculogenesis

**DOI:** 10.3389/fendo.2018.00134

**Published:** 2018-03-29

**Authors:** Sabine Mazerbourg, Philippe Monget

**Affiliations:** ^1^Université de Lorraine, CNRS, CRAN, Nancy, France; ^2^INRA, Physiologie de la Reproduction et des Comportements, UMR 6078 INRA-CNRS-Université de Tours, Nouzilly, France

**Keywords:** insulin-like growth factor, insulin-like growth factor binding protein, ovary, folliculogenesis, pregnancy-associated plasma protein-A, stanniocalcins

## Abstract

The aim of the present article is to update our understanding of the expression of the insulin-like growth factor binding proteins (IGFBPs), IGFBP proteases and their implication in the different processes of ovarian folliculogenesis in mammals. In the studied species, IGFs and several small-molecular weight IGFBPs (in particular IGFBP-2 and IGFBP-4) are considered, respectively, as stimulators and inhibitors of follicular growth and maturation. IGFs play a key role in sensitizing ovarian granulosa cells to FSH action during terminal follicular growth. Concentrations of IGFBP-2 and IGFBP-4 in follicular fluid strongly decrease during follicular growth, leading to an increase in IGF bioavailability. Inversely, atresia is characterized by an increase of IGFBP-2 and IGFBP-4 levels, leading to a decrease in IGF bioavailability. Changes in intrafollicular IGFBPs content are due to variations in mRNA expression and/or proteolytic degradation by the pregnancy-associated plasma protein-A (PAPP-A), and likely participates in the selection of dominant follicles. The identification of PAPP-A2, as an IGFBP-3 and -5 protease, and stanniocalcins (STCs) as inhibitors of PAPP-A activity extends the IGF system. Studies on their implication in folliculogenesis in mammals are still in the early stages.

Until the 2000s, involvement of insulin-like growth factors (IGFs) and IGF-binding proteins (IGFBPs) in ovarian folliculogenesis has been extensively described in several mammalian species (1). Comparative analysis reveals some species differences concerning the role of IGFs on the different stages of folliculogenesis, and on the changes of levels and expression of the different elements of IGF system during follicular growth and atresia. In all these species, IGFs and several small-molecular weight IGFBPs (in particular IGFBP-2 and IGFBP-4) are considered, respectively, as stimulators and inhibitors of follicular growth and maturation. Based on our complete review of the IGF system in the mammalian ovary (1), we will present an update on IGFBPs expression and IGFBP proteolytic degradation in the ovary, with a focus on the protease, pregnancy-associated plasma protein-A (PAPP-A).

## The IGF System

The IGF system is composed of different elements (2–4):

– Two ligands, IGF-I and IGF-II.
– Two receptors: the type I receptor mediates most of the somatomedin-like actions of both IGF-I and -II. The type II receptor, or IGF-II/Mannose-6-Phosphate (IGF-II/M6P) receptor, binds IGF-II but not insulin, and binds IGF-I with very low affinity, and can be considered as an inhibitor of IGF-II action.
– Six IGFBPs, which bind IGF-I and -II with high affinity. IGFBPs are present in all biological fluids. They can be arbitrarily classified in two groups: (1) the small molecular weight IGFBPs, or IGFBPs <40 kDa including IGFBP-1, -2, -4, -5, and -6 that are present in the serum and in other fluids in a so called “small complex.” When visualized by western-ligand blotting (WLB), their apparent molecular weights range between 24 and 35 kDa. In serum, their levels are either negatively regulated (IGFBP-1 and -2) or unaffected by growth hormone (GH); (2) IGFBP-3, which is the predominant IGFBP in serum. In this fluid, it is mostly present in a 150 kDa form (“large complex”), composed of IGF-I or IGF-II, and an acid-labile 85 kDa subunit (ALS). Native IGFBP-3 is visualized as a 44–42 kDa doublet by WLB. The concentration of IGFBP-3 is positively regulated by GH and IGF-I.

Insulin-like growth factor binding proteins increase IGFs half-life and constitute a large pool of IGFs in all the compartments of the organism (5, 6). Furthermore, IGFBPs can both inhibit and potentiate IGFs action at the level of target cells. They can indeed inhibit IGFs action by sequestration, since the affinity of IGFBPs for IGF-I and -II is of the same order of magnitude as the affinity of type I receptors. However, the affinity of IGFBPs for IGF-I and -II can be modulated by post-translational changes. Particularly, the affinity of IGFBP-1 for IGFs can be increased by phosphorylation as reported in human amniotic fluid at the end of pregnancy (2, 7, 8). Moreover, the affinity of IGFBPs for IGFs can be reduced when IGFBPs are bound to ECM (IGFBP-5), or when they are proteolyzed (IGFBP-2, IGFBP-3, IGFBP-5, and IGFBP-4) (3, 8). Such proteolysis has been first described in the serum of female rodents and humans during pregnancy, after nutritional fasting, during severe illness or following extensive trauma. Limited proteolysis of IGFBPs has also been described in the serum and lymph of normal human adults, and in culture media of osteoblastic cells, granulosa cells, and tumor cells (9). It is, therefore, likely that such a phenomenon exists *in vivo* in most tissues. Moreover, it is important to note that this proteolysis can occur at the level of the cell membrane (10). Such decreases in the affinity of IGFBPs for IGFs, hereby increasing bioavailability of IGFs, can lead to a potentiation rather than an inhibition of action of the ligands.

## IGFs in the Ovary

There are many *in vitro* and *in vivo* evidence that IGF-I and IGF-II are stimulators of ovarian follicular development (1, 11, 12). IGF-I stimulates either proliferation, or differentiation of granulosa cells depending on the stage of development of the follicle, and plays a key role in the responsiveness of the ovary to FSH action. Moreover, the increase in expression and/or bioavailability of IGFs in large preantral follicles results in an increase in the number of functional FSH receptors, leading to an increase in type I IGF receptors. This positive feedback loop might partly be responsible for the amplification of FSH action and the expression of aromatase and LH receptors in fully mature follicles. In contrast to adult rodents that have trace amounts of IGF-II in serum, adult humans (as well as adult sheep, cattle, and pigs), contain twofold to threefold more IGF-II than IGF-I in serum, the former being less dependent on GH than the latter. In cattle and mice, IGF-I seems to play a key role in increasing the sensitivity of small antral follicles to gonadotropin action, and plays a key role in their transition to the gonadotropin-dependent follicular stage. In human, circulating IGF-I does not seem to be essential for the development or maturation of ovarian follicles (13–15). In this case, it is possible that the low (likely GH-independent) expression of IGF-I in small growing follicles and of IGF-II in large dominant follicles are able to replace the contribution of serum GH-dependent IGF-I.

There is some heterogeneity in the localization of IGFs expression in the ovary of different species (1), but several arguments play in favor of a main seric origin of IGFs (16). Except in human, it is likely that small changes in local expression of IGFs would not have any significant consequence on their intrafollicular concentration, considering the high levels present in serum. Moreover in the dog, Reynaud et al. show that the wide span in body height among dogs with different breeds is associated with dramatic differences in IGF-I and IGFBP-3 levels in both plasma and follicular fluid from preovulatory follicles (17). These differences of levels impact follicular development: large dogs have a higher number of preovulatory follicles than small dogs, these follicles being 70% larger in the largest dog than in the smallest dog (17). These differences are not associated with differences in estradiol serum levels, suggesting an uncoupling between the number and the size of preovulatory follicles in this species, and steroidogenesis.

Actually, IGFs bioavailability, rather than IGFs concentration, dramatically changes during growth and atresia of ovarian follicles (see below).

## IGFBPs in the Ovary

The intrafollicular IGFBP content fluctuation is a very conserved phenomenon among mammalian species: the disappearance of IGFBPs < 40 kDa (IGFBP-2, IGFBP-4 as well as IGFBP-5 in ruminants follicles) characterizes the preovulatory follicles and the increase in their levels is observed in atretic follicles. All these changes are due to two processes: changes in mRNA expression, and changes in proteolytic degradation (Table 1).

**Table 1:** General overview of IGFBP contents variations during follicular growth and atresia in mammalian ovary.

	Growing follicles			Atretic follicles	
	mRNA	Protein	Proteolysis	Protein	Proteolysis
IGFBP-1		Mainly from serum	nd	nd	nd
IGFBP-2	↔/↓*	↓	↑	↑	0
IGFBP-3	↔/0*	↔ or mainly from serum*	nd	↔	nd
IGFBP-4	↓/↔*	↓	↑	↑	0
IGFBP-5	↓/↔/0*	↓/0*	↑	↑/↔/0*	0
IGFBP-6	↔/↑*	nd	nd	nd	nd

*This table summarizes global changes in follicular mRNA and protein expression levels of IGFBP-1 to -6 and variations in intrafollicular IGFBP proteolytic activity observed in most mammalian species. Species specificities are reported in the text*.

*, depending on the species; ↓, decrease; ↑, increase; ↔, no change; 0, absence; nd, not determined.

## Changes in mRNA Expression

### IGFBP-1

Most of the studies report the absence of significant expression of IGFBP-1 in mammalian ovaries (18–22). However, in human ovaries, some studies have shown IGFBP-1 mRNA expression in granulosa cells of mature follicles (23, 24). Overall, it could be postulated that in most mammalian species, intrafollicular IGFBP-1 derives mainly from serum, rather than *de novo* synthesis.

### IGFBP-2

In the ewe, the sow, the cow, and the mare, intrafollicular levels of IGFBP-2 strongly decrease from 1 to 2 mm diameter follicles to preovulatory follicles. By contrast, its intrafollicular level strongly increases in atretic follicles (1, 25–28). *In vivo*, a strong decrease in IGFBP-2 mRNA expression has been observed in granulosa cells during follicular growth in ovine (29), porcine (30, 31), bovine (32), brushtail possum (33), but not in primate and human ovaries (24, 34). By contrast, IGFBP-2 mRNA expression has been shown to increase in the same compartment and in theca cells of atretic follicles in sheep (29). In the brushtail possum, IGFBP-2 mRNA is also expressed in granulosa and theca cells of atretic antral follicles (33). So in several species, the decrease and the increase in IGFBP-2 mRNA expression during follicular growth and atresia, respectively, partly explains changes in intrafollicular levels of the protein. *In vitro*, FSH and/or cAMP strongly inhibit IGFBP-2 expression by granulosa cells in pigs (35, 36), in cattle (32), and humans (37), suggesting that the diminution of levels of this IGFBP in preovulatory follicles is FSH dependent (32). In the pig, the decrease in IGFBP-2 protein level is not associated with a decrease in mRNA levels (35, 36), suggesting that FSH could act through an increase in IGFBP-2 degradation *in vitro*.

### IGFBP-3

IGFBP-3 levels do not seem to change during folliculogenesis in any species. In ovarian follicles, expression of IGFBP-3 is low and poorly or not associated with growth or atresia (24, 38). *In vitro*, IGFBP-3 mRNA expression was positively regulated by FSH in small bovine granulosa cells and downregulated by estradiol in theca cells of large bovine follicles (39). Inversely, in cultured pig and human granulosa cells, FSH inhibited IGFBP-3 production (40, 41).

### IGFBP-4

Changes in IGFBP-4 intrafollicular levels are similar to those observed with IGFBP-2 with a decrease in growing follicles and an augmentation in atretic ones (1, 25–27). In contrast to IGFBP-2, expression of IGFBP-4 mRNA differs between species. In particular, IGFBP-4 mRNA levels are low in both granulosa and thecal cells of ovine and bovine species, and exhibit only slight changes during follicular growth and atresia (32, 42). In the particular case of cystic ovarian disease, one of the main causes of infertility in dairy cattle characterized by the persistence of large follicular structures, IGFBP4 expression *in situ* is higher in granulosa cells in persistent follicles than in control follicles (43). In human ovaries, IGFBP-4 mRNA content is high in immature follicles and dramatically decreases in mature follicles (24). In the brushtail possum, IGFBP-4 mRNA is limited to theca cells of large preantral and antral follicles (33). In the rat, IGFBP-4 mRNA expression strongly increases in granulosa cells of atretic follicles (44). This upregulation of IGFBP-4 mRNA in atretic rat follicles is likely due to their loss of FSH sensitivity. Indeed, *in vitro*, FSH was shown to strongly decrease expression of IGFBP-4 by rat granulosa cells (45). In sharp contrast, IGFBP-4 mRNA expression increases in thecal cells during follicular growth in the sow and the monkey (31, 46). In the latter species, 7 days treatment of hCG-induced a marked increase in IGFBP-4 mRNA expression detected by *in situ* hybridization in thecal cells *in vivo* (46). However, a more recent study reported a reduction of IGFBP-4 mRNA levels in monkey granulosa cells 24 h after injection of hCG (47).

*In vivo*, GnRHa injection in sheep, disrupting FSH and LH regulation, induces a significant decrease in IGFBP-4 mRNA expression after 36 h from start of treatment in large but not medium and small follicles (48). Interestingly, LH stimulates rather than inhibits IGFBP-4 expression by bovine thecal cells *in vitro* (32). These results are concordant with *in vivo* effects of hCG on IGFBP-4 expression in rhesus monkeys described by Zhou et al., as well as with data on the increase in IGFBP-4 mRNA expression in large preovulatory porcine follicles (31, 46). However, these observations clearly contrast with the fact that in all these species, IGFBP-4 protein levels are undetectable in the follicular fluid of these follicles. This is due to its proteolysis by the PAPP-A protease (see below).

### IGFBP-5

Ovarian expression of IGFBP-5 mRNA also strongly differs between species. This expression slightly decreases during follicular growth in ovine thecal cells, but dramatically increases in granulosa cells from ovine and rat atretic follicles (42, 44, 48). In bovine follicles, high levels of IGFBP-5 mRNA and protein are detected in subordinate follicles compared to dominant follicles (49). In sharp contrast, in the mouse, IGFBP-5 transcript levels are elevated in granulosa cells of healthy primary and secondary follicles, and decrease in subsequent follicular stages and in atretic follicles (50). In rhesus monkeys, IGFBP-5 mRNA is selectively expressed in thecal and granulosa cells of dominant but not immature follicles (34). In the pig, expression of IGFBP-5 mRNA is concentrated on the surface of the germinal epithelium and in capillary endothelium (20). Finally, in the brushtail possum, IGFBP-5 mRNA expression is limited to granulosa cells of primordial, primary, and some secondary follicles. Strong expression is also observed in the theca of secondary and antral follicles (33). As for IGFBP-4, FSH strongly inhibits IGFBP-5 mRNA expression by rat granulosa cells *in vitro* (45). By using a model of serum withdrawal-induced programmed cell death, we have shown that IGFBP-5 mRNA expression is enhanced in apoptotic ovine granulosa cells *in vitro*, suggesting that the expression of this IGFBP is associated with cell viability in this species (28). *In vivo*, in sheep, Hastie et al. disrupted FSH and LH regulation with a GnRHa injection, leading to a transient increase in IGFBP-5 mRNA expression after 12 h from start of treatment. These data suggest that gonadotrophins modulate IGFBP-5 expression (48).

### IGFBP-6

To our knowledge, IGFBP-6 intrafollicular content has never been documented. In monkey ovaries, IGFBP-6 mRNA was present at low levels in the interstitium and theca externa and was more abundant in the ovary surface epithelium (34). In bovine follicles, IGFBP-6 mRNA has been detected only in theca cells with a higher expression during the final follicular growth (21). In cycling ewes, IGFBP-6 mRNA expression does not change in small follicles, no matter their health status. However, its expression significantly decreases in large atretic follicles compared to large healthy follicles (22).

## Changes in Intrafollicular Proteolytic Degradation

Changes in IGFBP levels during folliculogenesis can partly be explained by changes in intrafollicular proteolytic activity. Indeed, Chandrasekher et al. have shown the presence of a proteolytic activity degrading IGFBP-4 in follicular fluid from human dominant estrogenic but not atretic follicles (51). Proteolytic degradation of IGFBP-4 and -5 was also maximal in ovine, bovine, porcine, as well as equine preovulatory follicles (42, 52–54). IGFBP-2 proteolysis was also detected in follicular fluid of preovulatory follicles of the different mammalian species (42, 52, 53, 55, 56). Nevertheless, *in vivo*, proteolytic degradation of IGFBP-2 likely occurs only in preovulatory follicles that exhibit a high IGF bioavailability, whereas IGFBP-4 is already degraded in healthy growing follicles. Interestingly, in the ewe, native IGFBP-4, assessed by WLB, was undetectable in follicles that contained more than 10 ng/ml estradiol. By contrast, native IGFBP-2 was undetectable only in follicles that contained more than 100 ng/ml estradiol, suggesting that the disappearance of IGFBP-2 during follicular growth occurs later than that of IGFBP-4 (1, 42). Besides, cleavage of IGFBP-2 was also observed after incubation with cultured mural granulosa cells from antral bovine follicles (57). No proteolysis was detected after incubation with denuded oocyte or oocyte cumulus complexes, suggesting that the bovine mural granulosa cells are the major source of the soluble protease (57). In these different conditions of culture, the addition of recombinant IGF-I or FSH had no effect on IGFBP-2 degradation rate (57).

## IGFBP Proteases in the Ovary

### Pregnancy-Associated Plasma Protein-A (PAPP-A)

Pregnancy-associated plasma protein-A is a member of the pappalysin family of metzincin metallo-proteinases (58). PAPP-A is a large dimeric glycoprotein of 400 kDa present in increasing concentrations in serum during human pregnancy. It circulates as a 2:2 disulfide bound complex of 500 kDa with the proform of eosinophil major basic protein (proMBP), denoted PAPP-A/proMBP (59, 60). No physiological function has been attributed to this circulating protein until Lawrence et al. showed that PAPP-A was the protease responsible for the proteolytic degradation of IGFBP-4 in human fibroblasts and osteoblasts cells-conditioned media (61). Then, PAPP-A was identified as the protease targeting IGFBP-4 in human, ovine, bovine, equine, and porcine follicular fluid (62–64). Thereafter, it was shown that IGFBP-2 was a substrate of PAPP-A in bovine, porcine, and equine preovulatory follicles (55, 56). In the mare, intrafollicular injection of PAPP-A into the second largest follicle (F2) decreases the concentration of IGFBP-2 to a level similar to the concentration in the largest follicle F1 (65). Finally, Rivera and Fortune have reported that IGFBP-5 is degraded by PAPP-A in bovine preovulatory follicles but not in subordinate follicles of the same cohort (64). Overall, these results suggest that degradation of IGFBP-2, IGFBP-4, and IGFBP-5 by PAPP-A in preovulatory follicles is a well-conserved mechanism in mammalian species. In bovine and equine ovary, PAPP-A, and the associated decrease in intrafollicular IGFBP-4 level, could be a key factor in the mechanism leading to selection of dominant follicles (56, 64). In female mice, the absence of PAPP-A results in altered fertility, associated with reduced estradiol levels and reduced ovulation (66). The ovaries exhibit a small size but all follicular stages are present. Follicular fluid from *Pappa* knockout (KO) mice is totally deprived of IGFBP-4 proteolytic activity, strongly suggesting that the altered reproductive capacity of these female mice is a consequence of reduced IGF bioavailability in the follicular compartment (66). Interestingly, this study highlights that the PAPP-A is the exclusive intraovarian IGFBP-4 protease in the mouse. Since PAPP-A tethers to the surface of cells by binding to surface glycosaminoglycans, this protease is believed to regulate IGF signaling locally in tissues by increasing the pericellular level of bioactive IGF (67, 68).

#### PAPP-A Expression Has Been Characterized in the Mammalian Ovary

Several studies analyzed PAPP-A mRNA expression in the mammalian ovary. In the bovine and porcine ovary, a first study reports that PAPP-A mRNA expression in granulosa cells was maximal in preovulatory follicles and positively correlated with expression of both aromatase and LH receptors (63). In a second study in bovine ovary, Sudo et al. did not observe a significant difference of PAPP-A mRNA expression levels between follicles at all development stage, but the expression showed a tendency to increase with follicular growth (69). In a third study, Santiago et al. reported neither a significant differential expression of PAPP-A mRNA in granulosa cells of dominant and subordinate bovine follicles, nor a correlation between levels of PAPP-A mRNA and estradiol levels in follicular fluid (49). In the rodent ovary, ovarian cells expressing detectable PAPP-A mRNA are the centrifugally located mural granulosa cells of healthy growing antral, as well as preovulatory follicles, and the lutein cells of the corpus luteum. Both PMSG and hCG strongly stimulate PAPP-A expression *in vivo* (66, 70, 71). By *in situ* hybridization in the human ovary, Hourvitz et al. have observed PAPP-A expression in preovulatory but not immature follicles, as well as in the corpus luteum (72). Recently, an immunohistochemical study on human ovaries revealed that PAPP-A expression is primarily observed in the theca cells of small antral follicles, then in both theca and (slightly) granulosa cells of antral follicles, and finally in granulosa cells of preovulatory follicles (73). In that case, PAPP-A is co-expressed with aromatase in granulosa cells of antral and preovulatory follicles. Furthermore, the intrafollicular PAPP-A concentration is strongly positively correlated with estrogen and progesterone secretion and inversely correlated with testosterone and androstenedione levels (73). To support these expression data, Jepsen et al. have shown that intrafollicular concentration of PAPP-A increases in human antral follicles after administration of hCG (74). Similarly, in the rhesus monkey, PAPP-A mRNA expression shows a peak of induction 3 and 6 h after hCG induction leading to an increase in intrafollicular PAPP-A protein concentration 24 h later (47). By contrast, Zhou et al. have shown by *in situ* hybridization that PAPP-A is expressed in granulosa cells of antral follicles of all sizes, but did not observe any correlation with LH-receptor expression (46). Most of these results suggest a role for gonadotropin-stimulated PAPP-A gene expression in the shift from an androgenic to an estrogenic environment, characteristic of the follicular selection, as well as in the ovulation and luteogenesis processes in the mammalian ovary. However, we have to emphasize that ovarian PAPP-A protein level may not be systematically linked to IGFBP proteolytic activity. Indeed, in the human ovary, PAPP-A activity dramatically decreases in follicular fluid post-hCG treatment even though the intrafollicular concentration of PAPP-A was still maintained at a high level (74).

*In vitro*, Liu et al. and Resnick et al. have shown that FSH is able to induce the degradation of both IGFBP-4 and IGFBP-5 by rat granulosa cells-derived proteases (45, 75). Later on, PAPP-A was identified as the protease responsible for degradation of IGFBP-4 in FSH-primed rat granulosa conditioned media (71). Interestingly, the oocyte-derived bone morphogenetic protein-15 inhibits FSH-induced PAPP-A gene expression in rat mural and cumulus granulosa cell cultures. This result is likely to explain the absence of PAPP-A mRNA expression in the cumulus granulosa cells *in vivo* (see above) (71). In bovine granulosa cell cultures, Sudo et al. further report that PAPP-A mRNA is induced by FSH, and estradiol amplifies this hormonal stimulation (69). In humans, Conover et al. have shown that PAPP-A is secreted *in vitro* by granulosa cells from estrogen- but not androgen-dominant follicles (76). However, they did not observe any effect of hCG on PAPP-A levels in cell-culture medium.

Overall, these data suggest that PAPP-A is a granulosa cells-derived protease in the ovary and a marker of follicle selection and corpus luteum formation. The regulation of its expression highly depends on gonadotropins FSH or hCG/LH in most mammalian species. The involvement of intra-ovarian factors remains to be elucidated.

#### Intrafollicular PAPP-A Proteolytic Activity Is Modulated by IGFs

In ovine, bovine, and equine preovulatory follicular fluid, cleavage of IGFBP-4 and IGFBP-2 is enhanced in the presence of IGF-I and IGF-II (55, 56, 63). However, one can note that, in contrast to IGFBP-4, bovine and porcine preovulatory follicular fluids only induce a partial proteolytic degradation of exogenous IGFBP-2 in the absence of exogenous IGFs with this degradation being clearly enhanced in the presence of exogenous IGFs (55). The enhancing effect of IGF is confirmed by different biochemical analyses, suggesting that it is due to a conformational change of IGFBP-4 and -2 after IGF binding, and not to the binding of IGF to PAPP-A (77–80). The cleavage of IGFBP-5 by PAPP-A does not require the presence of IGF, but is slightly inhibited by IGF (78, 79). Thus, the concentration of IGF is likely to control the dynamics of proteolysis of IGFBP-2, -4, and -5 by PAPP-A.

#### Intrafollicular PAPP-A Proteolytic Activity Is Not Modulated by Pro-MBP but by Stanniocalcins (STCs)

As stated above, PAPP-A circulates in pregnancy serum as a disulfide-bound complex with proMBP that is able to strongly inhibit PAPP-A activity. *In vitro*, Conover et al. failed to detect any proMBP in human granulosa cell-conditioned media (76). Since then, STCs 1 and 2 (STC1 and STC2) have been presented as regulators of PAPP-A activity (81, 82). STC1 binds PAPP-A non-covalently but with high affinity, whereas STC2 requires covalent binding between the two proteins (81, 82). The stable covalent complex STC2/PAPP-A inhibits PAPP-A proteolytic activity toward IGFBP-4. In mice, transgenic overexpression of STC2, but not of the mutated proteolytically inactive STC2, induces a reduction in size of up to 45% (81, 83). A similar phenotype is observed in transgenic mice overexpressing STC1 as well as in *Pappa* and *Igf2* KO mice (84–86). The excess of STCs could neutralize PAPP-A activity leading to IGF sequestration by IGFBP-4 and a reduced local IGF bioavailability. Before the discovery of their new function, STC1 and STC2 expression had been described in some mammalian species. In the porcine ovary, STC1 protein was detected in theca and granulosa cells of growing follicles, in the oocyte and in luteal cells (87). A similar pattern of expression of STC1 protein has been observed in the mouse, but with the absence of expression in granulosa cells (88, 89). In this species, STC1 mRNA expression increases significantly after hCG treatment (89). In the rat, STC1 transcripts are localized in theca cells and are downregulated after PMSG treatment. After hCG induction, their levels remain low in the theca cells and in the corpora lutea, which contrasts with data in the mouse (89, 90). Conversely, STC2 mRNA expression is limited to theca cell layer of rat antral and preovulatory follicles after PMSG stimulation. Its level keeps decreasing after hCG treatment (91). In cultured early antral follicles, STC2 transcripts are induced by estrogen (91). In granulosa cell cultures, STC1 and STC2 were shown to function as paracrine factors suppressing FSH-induced progesterone production (90, 91). Overall, in the rat, due to the low expression of STC1 in the PMSG-primed follicles, STC2 is probably the STC isoform playing a role in the inhibition of luteinization of preovulatory follicles (91). In humans, in contrast to other species in which the STCs are expressed mainly in the theca cells, STC1 and STC2 are coexpressed in the granulosa cells and/or the theca-interstitial cells with variable intensity depending on the follicles (74). These authors showed a similar profile of expression with PAPP-A in primordial, late primary, antral follicles, and in the oocyte. The three proteins are consistently co-expressed in the same cell type during the process of follicle maturation, suggesting that they are able to form functional complexes (74). Indeed, PAPP-A:STCs complexes were immunoprecipitated in human follicular fluid, showing for the first time the physiological existence of these complexes (74). The presence of STCs could explain the decrease of PAPP-A activity observed in follicular fluid post-hCG treatment although the intrafollicular concentration of PAPP-A was still maintained at a high level (74).

Several points remain to be answered: (1) STCs are co-expressed with PAPP-A in human growing follicles while PAPP-A activity is high. Could STCs function/activity be controlled by another partner? (2) current studies suggest that the intra-ovarian STCs expression is not well conserved among species. More studies need to be done on the STCs protein expression rather than mRNA expression. Indeed, Varghese et al. have reported a different profile of expression in the mouse ovary between *in situ* hybridization and immunohistochemistry (88); (3) how is regulated the formation of the intrafollicular complexes PAPP-A:STCs? One could suggest that the expression level of each partner could influence their association rate; and (4) more studies are needed to define whether STC1 and/or STC2 are the physiological regulators of the PAPP-A activity. STC1 and STC2 belong to the same family but their protein sequences show only 30% identity (91). Due to the presence of additional disulfide bridges, STC2 could have a different tertiary structure compared to STC1 (91). However, both recombinant proteins STC1 and STC2 form disulfide-linked homodimer or an inhibitory complex with recombinant PAPP-A (74, 82, 91). Moreover, both STC1 and STC2 are expressed in human developing follicles, and are present in complexes with PAPP-A in follicular fluid (74, 82). At this time, it is impossible to delineate the relative importance of STC1 compared to STC2 in PAPP-A inhibition; (5) The exact role of STCs in the ovary has to be characterized. STC1 and STC2 have been presented as homodimeric ligands inhibiting FSH stimulation of rat granulosa cells differentiation through activation of specific receptors (90, 91), but also as the modulators of the IGF system (74). In this context, studies on mice with ovarian invalidation of *Stc1* and/or *Stc2* genes would be very informative.

### Pregnancy-Associated Plasma Protein-A2 (PAPP-A2)

Pregnancy-associated plasma protein-A2, a paralog of PAPP-A, is responsible for the IGF-independent proteolytic degradation of IGFBP-5 and IGFBP-3 (92, 93). Both STC1 and STC2 can inhibit the activity of PAPP-A2, blocking the release of IGFs from IGFBP-3 and IGFBP-5 (82, 94). Homozygous loss-of-function mutations in PAPP-A2 result in a novel syndrome of growth retardation with markedly elevated circulating IGF-I and IGF-II, but a decrease of bioactivity due to an increase in serum concentrations of IGFBP-3 and -5 sequestrating IGFs (93). In *Pappa2* KO mice, ovarian IGFBP-5 expression increases, but *Pappa2* deletion does not affect female reproduction (95). This phenotype does not mimic the complex phenotype of *Igfbp-5* overexpressing transgenic mice showing reduced female fertility (96). The expression profile and the local action of ovarian PAPP-A2 during folliculogenesis remain to be evaluated.

## Conclusion

**Figure 1:**
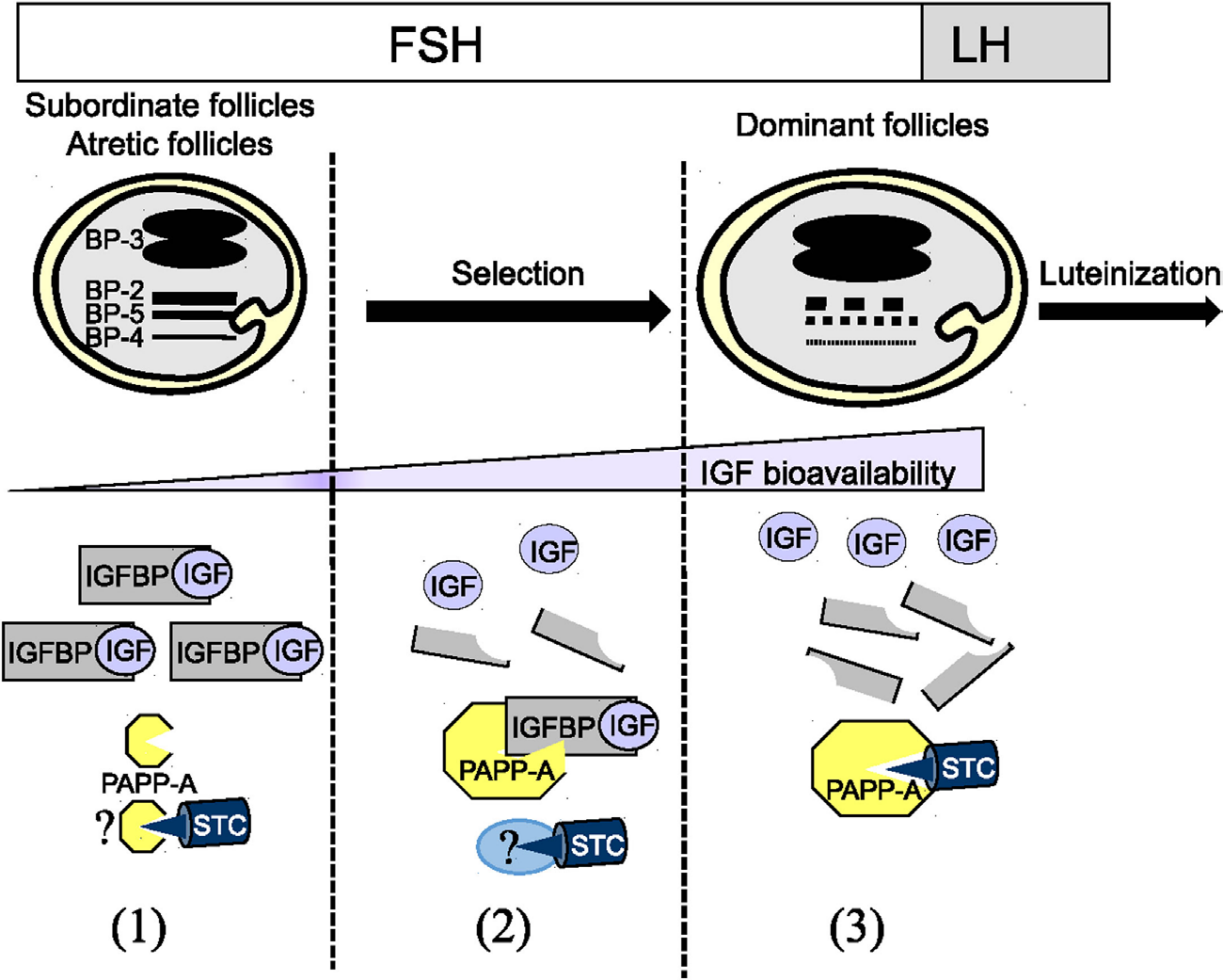
Dynamic of the insulin-like growth factor binding protein (IGFBP) system during folliculogenesis (1) High concentrations of IGFBP-2, IGFBP-4, and IGFBP-5 in follicular fluid of subordinate and atretic follicles lead to a low IGF bioavailability. The absence of IGFBP proteolytic degradation is due to the low expression level of PAPP-A and potentially to its association with the inhibitor STCs STC1/2. (2) During terminal follicular growth under FSH control, the decrease in IGFBP-2, IGFBP-4, and IGFBP-5 is due to a decrease in IGFBP mRNA expression and an increase in the proteolytic degradation by PAPP-A in follicular fluid. This decrease might participate in the selection of dominant follicles. At this stage, PAPP-A expression and activity are maximal. Surprisingly, the inhibitor STC1/2 is coexpressed with PAPP-A suggesting that STC1/2 activity could be neutralized by a partner to allow PAPP-A full activity. (3) Just before ovulation, after LH stimulation, PAPP-A activity is reduced likely through its association with STC1/2.

In the last two decades, studies on IGFBPs and folliculogenesis have been revisited with the identification of IGFBP proteases, PAPP-A and PAPP-A2, and more recently by the PAPP-A inhibitors STCs. The dynamics of ovarian IGFBP and PAPP-A concentrations has been linked to the selection of dominant follicles. However, much is yet to be learned on the role of STCs in this process (Figure 1).

Most of the studies on ovarian folliculogenesis attribute an inhibitory function to IGFBPs by sequestrating IGFs. However, it is well documented that IGFBPs could favor a local action of IGFs by concentrating the ligand in the vicinity of the IGF receptor (97, 98). Indeed, deletion of IGFBP-4 in *Igfbp-4* null mice results in growth retardation, suggesting that loss of IGFBP-4 leads to the loss of its pericellular reservoir function (99). A similar phenotype is observed in *Pappa* null mice with accumulation of IGFBP-4. In this case, IGFBP-4 is likely to sequester IGF-II, prevent its release, and abolish most of IGF-II-stimulated growth. Then, PAPP-A could potentiate IGF action through its binding to the cell surface to target IGF-II/IGFBP-4 complex to the vicinity of the IGF receptor. Thus, these models of null mice highlight that IGFBP-4 can have both stimulatory and inhibitory effects on growth and point out the absence of any redundancy between IGFBPs in this context (99). What about in the ovary? KO mice lacking either IGFBP-2, -3, -4, or -5 and the triple KO IGFBP-3/-4/-5 mice are still fertile suggesting redundancy between IGFBPs and compensatory mechanisms (100, 101). Indeed, in *Igfbp-2* null mice, seric IGFBP-1, IGFBP-3, and IGFBP-4 levels are increased relative to wild-type mice (100). To our knowledge, no data are available on changes of intrafollicular IGFBP protein content. Complementary data come from the study on *Pappa* KO model showing a lower fecundity (reduction of the number of ovulated oocytes and the litter size), with no alteration of follicular growth (normal histology) (66). In this context, follicular fluid is deprived of IGFBP-4 proteolytic activity suggesting the accumulation of IGFBP-4. It would be of interest to know whether high intrafollicular IGFBP-2 and -5 levels are also maintained in the absence of PAPP-A. Nevertheless, this study reveals that limiting intrafollicular IGFBP proteolysis may decrease IGF bioavailability in growing follicles, but not enough to block folliculogenesis as observed in *Igf1* null mice (11). Potential compensatory mechanisms could be initiated such as reduction of expression of IGFBP-2, -3, -4 or activation of other IGFBP-2 and -5 proteases (66). Overall, in the mouse ovary, these results strengthen the inhibitory role of intrafollicular IGFBPs.

Insulin-like growth factor (IGF)-independent functions of IGFBPs have not been really tackled in the ovarian follicle context. In cancer cells, the interaction of IGFBPs with non-IGF ligands, the intracytoplasmic and nuclear action of IGFBPs have been reported (97, 98, 102). The contribution of the non-canonical function of IGFBPs remained to be investigated on ovarian cells.

## Author Contributions

SM and PM contributed to the redaction of the review.

## Conflict of Interest Statement

The authors declare that the research was conducted in the absence of any commercial or financial relationships that could be construed as a potential conflict of interest.
